# Supplementary material for: Cerebrospinal Fluid from Healthy Pregnant Women Does Not Harbor a Detectable Microbial Community
Source: Microbiol Spectr. 2021 Dec 15;9(3):e00769-21. doi: 10.1128/Spectrum.00769-21 (PMC8672898; doi:10.1128/Spectrum.00769-21)
Supplement: SUPPLEMENTAL FILE 1 — Supplemental material. Download SPECTRUM00769-21_Supp_1_seq2.pdf, PDF file, 0.5 MB [file spectrum00769-21_supp_1_seq2.pdf]

Supplemental Information

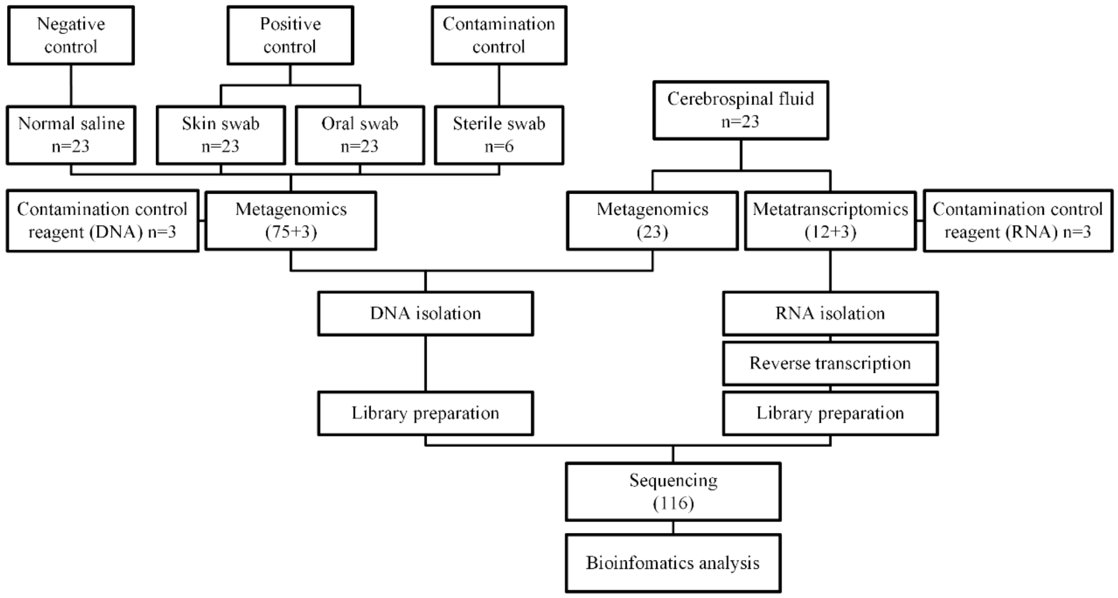

Fig. S1. Flow-chart to illustrate the study design and sample composition. CSF and matched control specimens (positive controls: oral and skin; negative controls: saline solution) collected from 23 pregnant women along with sterile swabs and DNA/RNA extraction buffers (contamination controls) were sequenced for metagenomic and metatranscriptomic analysis.

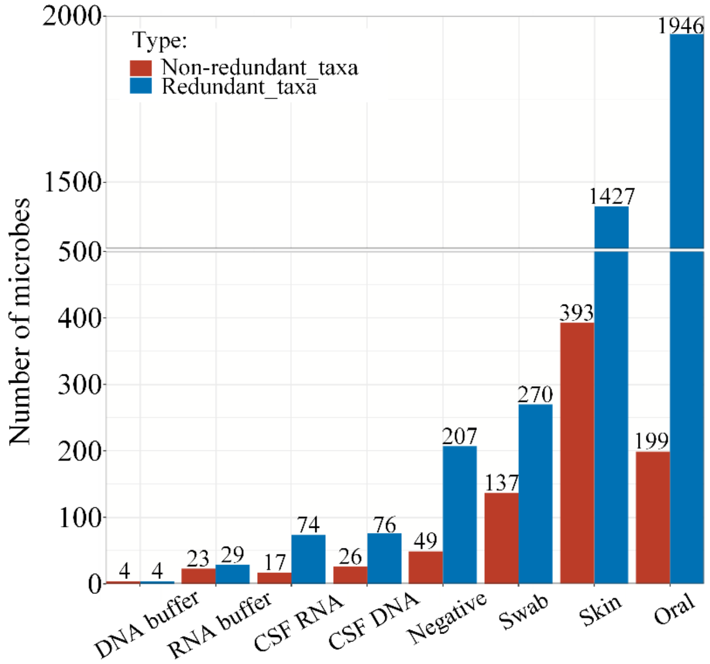

Fig. S2. Comparison of the number of redundant and nonredundant microbes in different types of specimens. The number of redundant and nonredundant microbial taxa respectively represents the cumulative total number of species detected and the total number of different types of species detected in a certain specimen type.

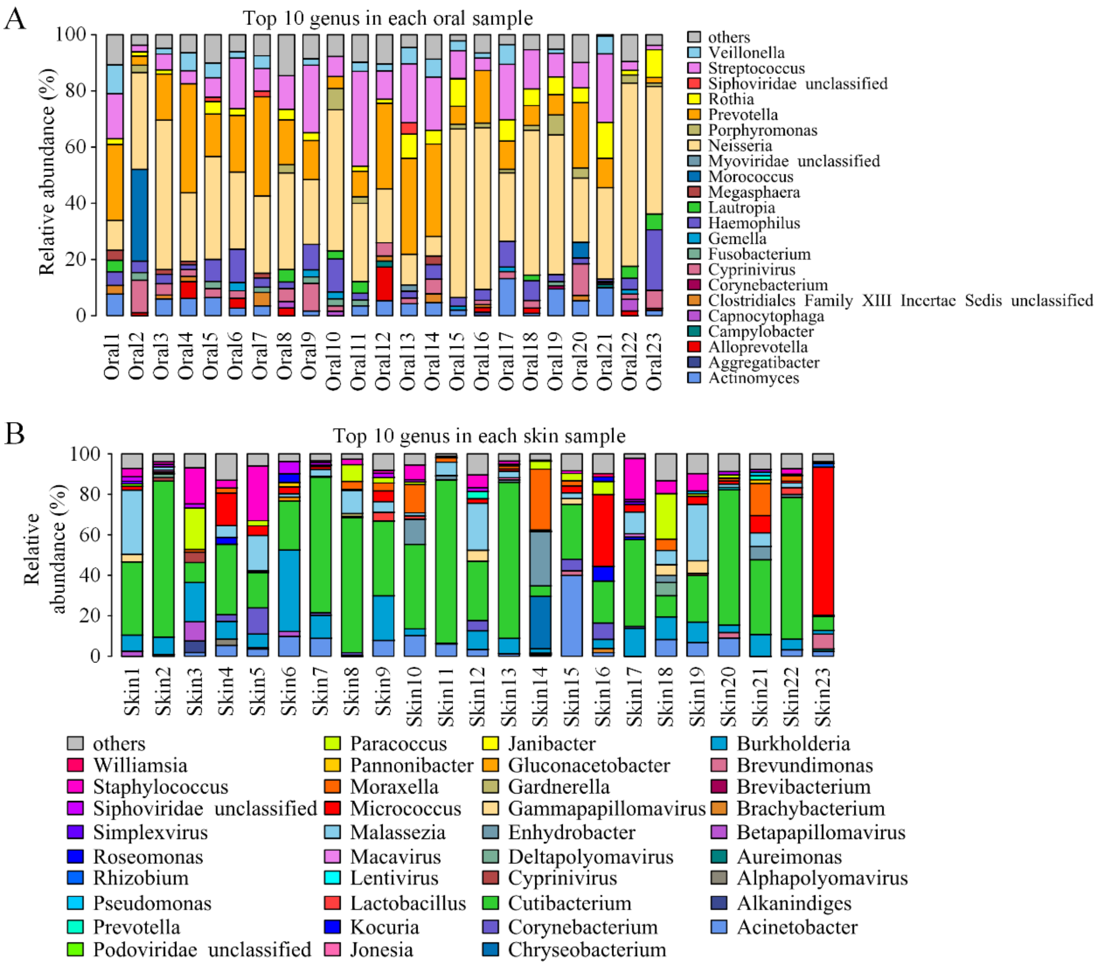

Fig. S3. Top 10 genus in oral and skin specimens, respectively. Microbial community structures of 23 Oral (Fig. S3A) and Skin (Fig. S3B) samples shown in a stacked barplot that summarizes the relative abundance of different genus detected.

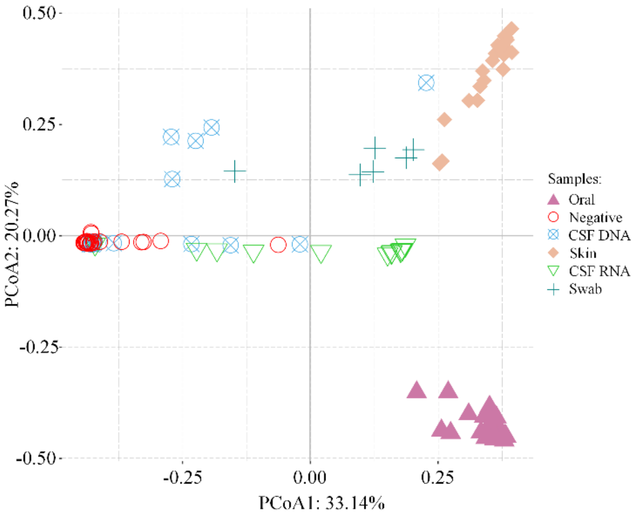

Fig. S4. Principal Coordinates Analysis (PCoA) analysis of microbial species detected from different types of specimens. Shapes and colors represent sample types.

Table S2: Specimen labels and matching information.

CSF DNA	Negative	Skin	Oral	CSF RNA	Swab	RNA buffer	DNA buffer
1	1	1	1	1	1	1	1
2	2	2	2		2	2	2
3	3	3	3		3	3	3
4	4	4	4	4	4		
5	5	5	5		5		
6	6	6	6		6		
7	7	7	7				
8	8	8	8	8			
9	9	9	9	9			
10	10	10	10	10			
11	11	11	11				
12	12	12	12				
13	13	13	13				
14	14	14	14	14			
15	15	15	15	15			
16	16	16	16	16			
17	17	17	17				
18	18	18	18	18			
19	19	19	19	19			
20	20	20	20				
21	21	21	21	21			
22	22	22	22				
23	23	23	23	23			

CSF DNA: metagenomic sequencing of cerebrospinal fluid; CSF RNA: metatranscriptomic sequencing of cerebrospinal fluid; Negative: normal saline specimens; DNA buffer: DNA extraction buffer specimens; RNA buffer: RNA extraction buffer specimens.
